# Outcome After Anterior Cervical Decompression and Fusion—A Nationwide FinSpine Register Study of Independent Predictors of Outcome at 12 Months After Surgery for Degenerative Cervical Spine

**DOI:** 10.1097/BRS.0000000000005323

**Published:** 2025-03-06

**Authors:** Nikolai Klimko, Nils Danner, Henri Salo, Anna Kotkansalo, Ville Leinonen, Jukka Huttunen

**Affiliations:** a Department of Neurosurgery, Kuopio University Hospital and Institute of Clinical Medicine, University of Eastern Finland, Kuopio, Finland; b Finnish Institute for Health and Welfare, Helsinki, Finland; c Department of Neurosurgery, Division of Clinical Neurosciences, Turku University Hospital, Turku, Finland

**Keywords:** ACDF, anterior cervical decompression and fusion, FinSpine, register study, independent predictors of outcome, degenerative cervical spine, DCSD

## Abstract

**Study Design.:**

Longitudinal, nationwide register study.

**Objective.:**

To identify independent predictors of clinical outcomes at 12 months for patients undergoing primary anterior cervical discectomy and fusion (ACDF) for degenerative cervical spine disease (DCSD).

**Summary of Background Data.:**

ACDF is an established surgical treatment for DCSD. Identifying factors that predict successful surgical outcomes can improve patient selection and inform decision-making.

**Methods.:**

This study utilized data from the Finnish National Spine Register (FinSpine), covering all Finnish centers that perform ACDF surgery. Patients undergoing primary ACDF surgery for DCSD between June 2016 and February 2024 without prior cervical spine surgery were included (n=5517). Patients were grouped based on the patient symptom status (“improved” *vs.* “indifferent or worse”) at 12 months postsurgery. Predictive factors were identified using classification tree analysis followed by binary logistic regression.

**Results.:**

At 12 months, 76.8% (n=1799) of patients reported symptom improvement, while 23.2% (n=542) reported that symptoms were indifferent or worse. Loss to follow-up for the outcome variable was 57.6% at 12 months. The following factors were associated with better outcomes: shorter preoperative pain duration (≤1 yr, OR=1.95, *P*<0.001), lower preoperative Neck Disability Index (NDI) scores (≤42, OR=1.37, *P*=0.012), and nonsmoking (OR=1.37, *P*=0.030). The initial diagnosis also influenced outcomes: patients treated for herniated disks and nerve root stenosis were more likely to report improvement compared to those with central canal stenosis or myelopathy (*P*<0.001). Gender, age, BMI, working status, regular use of pain medication, perioperative complications, muscle weakness, levels fused, and use of plate versus stand-alone cage were not independently predictive of outcomes.

**Conclusions.:**

Shorter preoperative pain duration, lower NDI scores, and nonsmoking status were significant predictors of good outcomes at 12 months after ACDF surgery for DCSD. These findings can help to guide preoperative patient counseling and enhance evidence-based decision-making for treating DCSD.

Degenerative cervical spine disease (DCSD) has become a significant socioeconomic burden, and its prevalence is increasing around the globe.^1,2^ In Finland, cervical disk herniations alone account for over 100,000 annual sick leave days in a population of 5.5 million.^3^ In the most recent disease burden report by the World Health Organization in 2021, neck and back pain caused the most disability-adjusted life years (DALYs) in the Finnish population aged 40 to 54 years.^4^

Most symptoms of DCSD can often be managed conservatively.^5–7^ However, while there is a group of patients for whom surgery is indicated, identifying those who will benefit most from surgical intervention remains controversial.^5–8^ Anterior cervical discectomy and fusion (ACDF), initially described by Cloward *et al*,^9,10^ in 1958, has since established its role as a main surgical treatment option for DCSD.^11,12^ ACDF can be used to treat both degenerative radiculopathy and myelopathy, regardless of whether the underlying cause is cervical disk herniation or degenerative spondylosis.^5,6,8^

Previous studies have addressed independent factors that may predict clinical improvement or nonsuccess after ACDF for DCSD.^13–16^ In this study, we aimed to find patient-related and treatment-related factors that predict outcomes after ACDF in a nationwide cohort of consecutive patients. The novelty of our study is the utilization of consecutive patient data from the Finnish nationwide spine register (FinSpine). The main question was: Who is most likely to benefit from ACDF surgery?

## MATERIALS AND METHODS

### FinSpine Register

FinSpine, the Finnish national quality register for spine surgery, contains comprehensive data from spine surgeries performed in Finland since 2016.^17^ Based on Finland’s tax-funded, universally accessible health care system, most spine surgeries are carried out in public hospitals. The current version of the register covers all 23 Finnish public hospitals as well as all major private hospitals.^17^

The register is maintained by the Finnish Institute for Health and Welfare, an independent agency under the Ministry of Social Affairs and Health, which is responsible for pooling and storing the data from all participating centers. In addition to maintaining a national quality register, the Finnish Institute for Health and Welfare aims to further develop the register and facilitate clinical research in collaboration with researchers and clinical experts.

FinSpine is designed to automatically collect data from the electronic patient records of participating hospitals as well as patient-reported baseline and outcome data along with surgeon-reported details of the procedures.^17^ Surgeon-reported parameters include: type of surgery (primary *vs.* secondary *vs.* revision), levels of fusion and decompression, fusion materials, in-hospital and late-occurring complications, diagnosis, muscle weakness caused by nerve root or spinal cord compression, and antibiotic and thrombosis prophylaxis. Relevant medical history, patient-reported outcome measures (PROMs), and patient-reported experience measures (PREMs) are collected directly from patients. Data collection includes visual analog scales (VAS; 0–100), the Neck Disability Index (NDI),^18^ Oswestry disability index (ODI),^19^ and EQ-5D.^20^ Patient-reported data are gathered by prompting patients to fill out questionnaires via an online platform. Automatic prompts are sent before the surgery and at 3 months, 1 year, 2 years, 5 years, and 10 years postoperatively. Participating hospitals have appointed designated research coordinators, who monitor the response process, provide assistance and send reminders as needed.^17^

### Study Setting and FinSpine Cohort

**Figure 1:**
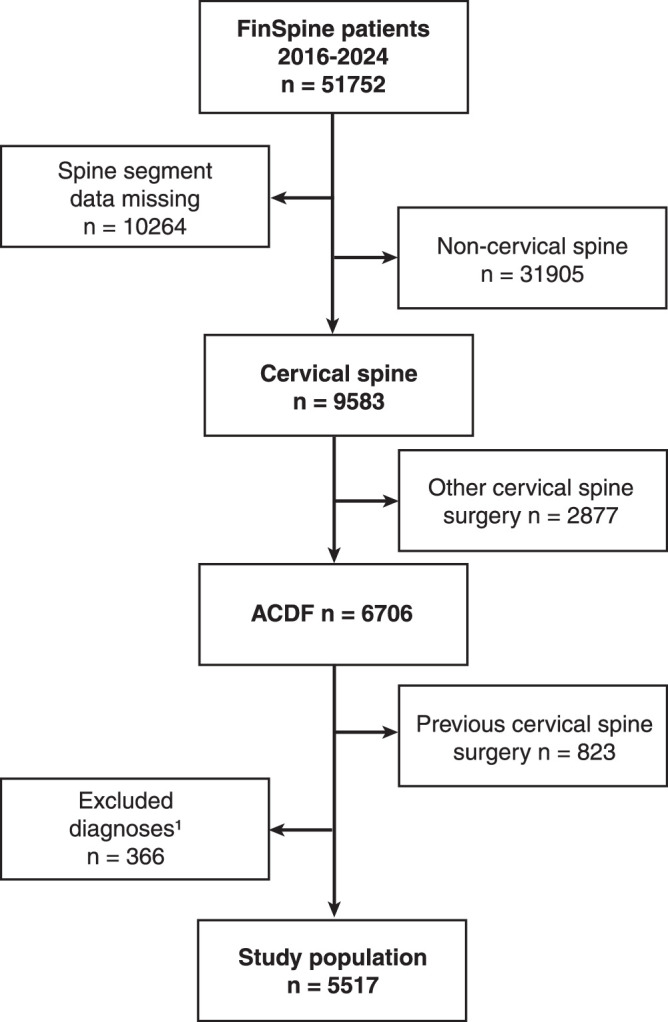
Flowchart of the FinSpine register population. The study population included patients who underwent primary ACDF for degenerative cervical spine disease. ^1^Nondegenerative and missing surgical diagnoses were excluded from the study population.

A flowchart of the study is presented in Figure 1. The current version of FinSpine contains records of patients who underwent spine surgery between June 9, 2016, and February 29, 2024. At the time of data extraction, FinSpine had entries from 19 out of 23 public hospitals, covering every center in Finland in which ACDF surgery is performed.

For this study, we included patients who underwent primary ACDF surgery during the study period and had no history of prior cervical spine surgeries. Patients were required to have one of the following diagnoses as the indication for surgery: disk hernia, nerve root stenosis, central canal stenosis, myelopathy, or degenerative disk disease. Accordingly, patients with any other diagnosis were excluded: trauma (n=269), metastasis (n=2), kyphosis (n=2), postoperative instability (n=1), primary infection (n=7), isthmic spondylolisthesis (n=39), and indication marked as “Other” (n=2). Cases in which the indication was missing (n=33) were also excluded.

Out of the 51,752 identified patients, ACDF was performed on 6706 patients, of whom 5517 met the inclusion criteria and formed the final study population.

### The Main Outcome Variable

The patient-reported experience measure of global perceived effect (GPE) at 12 months after surgery was chosen as the main outcome variable. GPE was selected as our primary outcome measure due to its simplicity and direct clinical relevance. GPE captures a broad sense of patient-perceived improvement that is relatively easy to interpret. The specific question posed was, “How are your symptoms currently?” with answer options being “improved,” “indifferent,” or “worse” compared with the situation before surgery. For all analyses, the outcome variable was grouped into two categories: the “Improved” group and the “Indifferent or Worse” group.

**Figure 2:**
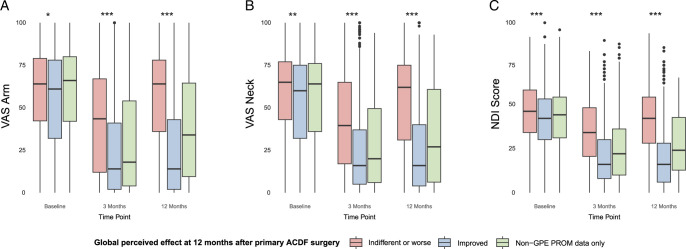
Patient-reported outcome measures in the primary ACDF population. Horizontal lines in boxplots represent median values. Statistical significance between “improved” and “indifferent or worse”—groups is presented with *—symbols. * *P*≤0.05, ** *P*≤0.01, *** *P*≤0.001. *P-*values were obtained using linear mixed models. “Non-GPE PROM data only”—group represents a group of patients for whom the global perceived effect (GPE) outcome variable data was lost to follow-up. NDI Score indicates Neck Disability Index Score; VAS, visual analog scale.

Whilst GPE may become more subjective over time and can be influenced by multifactorial issues,^21^ we complemented and validated our GPE data with the VAS and NDI scores to provide a more comprehensive outcome profile (Figure 2).

### Potential Predictive Baseline Variables

The selection of potential predictors for this study was primarily based on the variables available within the FinSpine register. FinSpine features an advisory group, comprising experts in the field, which has deliberated on and designed the national register.^17^ The variables used in FinSpine are similar to and even compatible with those used in other Nordic and European spine registers.^22–24^

Potential predictive baseline variables were: age; sex (male/female); body mass index (BMI); NDI score (0–100); separate VAS scores (0–100) for arm and neck pain; smoking status (yes/no); working status (working, unable to work, retired, unemployed); categorical pain duration (<6 wk, 6–12 wk, 3–12 mo, over 1 yr); regular use of pain medication (yes/no); diagnosis (disk herniation, nerve root stenosis, central canal stenosis, myelopathy, degenerative disk disease); muscle weakness due to nerve root or spinal cord compression (yes/no); perioperative complication (yes/no); levels fused (1–4); and was the fusion done with stand-alone cage or was a plate used.

### Statistical Analysis

Statistical analyses were performed using RStudio (version 2024.04.2) and IBM SPSS Statistics (version 28).

Descriptive data for the study population are presented according to the outcome variable, with means for continuous variables and counts for proportions. Changes over time in PROM results at three time points were estimated using linear mixed models (LMM).

To identify possible predictive factors, a classification tree analysis was conducted using the Chi-squared automatic interaction detection (CHAID) method, with parent and child node sizes of 250 and 100 subjects, respectively. Statistically significant factors (*P*<0.05) from the classification tree were then used to build a binary logistic regression model to estimate the possible independent effects of these factors. The regression model was internally validated by bootstrapping with 1000 resamples.

### Ethical Approval

This registered study has been permitted by the Finnish Institute for Health and Welfare and approved by the Ministry of Social Affairs and Health of Finland. Record number: THL/216/6.02.00/2024. According to Finnish research legislation, register-based studies do not require separate institutional ethical board approval.

## RESULTS

In the study population comprising 5517 patients undergoing ACDF surgery, the mean age was 52.8 years (SD±10.8), with a slightly higher proportion of males (52.0%, n=2868). Synthetic implants were used in all cases. A stand-alone cage was utilized in 98.8% of the cases (n=5545). The usage of a plate increased significantly with the number of fused levels: 7% for three levels, 1.2% for two levels, and 2.3% for a single-level fusion (*P*=0.018). The baseline characteristics of the study population are summarized in Table 1. The mean baseline NDI score was 42.7 (±17.1), with a VAS score for arm pain of 57.5 (±27.2) and a VAS score for neck pain of 55.5 (±26.9). Boxplots of baseline, 3-month, and 12-month VAS arm, VAS neck, and NDI scores are shown in Figure 2 (see Table, Supplementary Digital Content 1, http://links.lww.com/BRS/C655, for full descriptive statistics specific to the boxplots).

**TABLE 1:** Baseline Characteristics of the Study Population.

Variable	n	Missing, n (%)
Patients, n	5517	0
Age, mean (±SD)	52.8±10.8	0
Male, n (%)	2868 (52%)	0
BMI, mean (±SD)	28.6±5.3	2503 (45.4)
NDI Score, mean (±SD)	42.7±17.1	2600 (47.1)
VAS Arm, mean (±SD)	57.5±27.2	2657 (48.2)
VAS Neck, mean (±SD)	55.5±26.9	2594 (47.0)
Smoking, n (%)	739 (24.5)	2497 (45.3)
Working status, n (%)		2517 (45.6)
Working	1622 (54.1)	
Unable to work	741 (24.7)	
Retired	452 (15.1)	
Unemployed	185 (6.2)	
Pain duration, n (%)		2531 (45.9)
Less than 6 wk	140 (4.7)	
6–12 wk	264 (8.8)	
3–12 mo	1165 (39)	
Over 1 yr	1417 (47.5)	
Regular use of painkillers, n (%)	1763 (58.8)	2521 (45.7)
Diagnosis, n (%)		0
Disk hernia	1743 (31.6)	
Nerve root stenosis	2827 (51.2)	
Central canal stenosis	675 (12.2)	
Myelopathy	185 (3.4)	
Degenerative disk disease	87 (1.6)	
Radiculopathy, n (%)	1449 (26.7)	91 (1.6)
Spinal cord injury, n (%)	485 (8.9)	92 (1.7)
No. levels, n (%)		2964 (53.7)
One	1745 (68.3)	
Two	749 (29.3)	
Three	57 (2.2)	
Four	2 (0.1)	
Fused levels, n (%)		
C3-4	103 (3.0)	
C4-5	426 (12.5)	
C5-6	1530 (45.0)	
C6-7	1228 (36.1)	
C7-Th1	113 (3.3)	
Plate *vs.* stand-alone cage		0
Stand-alone cage	5453 (98.8)	
Cage + plate	64 (1.2)	
Perioperative complication, n (%)	90 (1.7)	130 (2.4)
Global perceived effect at 12 mo post-op, n (%)		3176 (57.6)
Improved	1799 (76.8)	
Indifferent or worse	542 (23.2)	

NDI Score indicates Neck Disability Index Score; VAS, visual analog scale.

The 12-month postoperative response rate for the GPE outcome variable in the cohort was 42.4% (n=2341). Improvement was reported by 76.8% (n=1799) of patients, whereas 23.2% (n=542) reported that their symptoms were indifferent or worse.

In the “Improved” group, the baseline NDI score was 41.8 (±0.5), with a significant reduction of 23.0 points (±0.4, *P*<0.001) at 12 months, as estimated by the linear mixed model. Similarly, the VAS arm pain score was 55.2 (±0.8) at baseline, with a 12-month reduction of 30.7 points (±0.9, *P*<0.001). The baseline VAS score for neck pain was 53.4 (±0.7), which declined by 29.2 points (±0.8, *P*<0.001) at 12 months. Patients who reported their symptoms as “indifferent or worse” at 12 months had higher baseline scores: NDI=46.4 (*P*<0.001), VAS arm=58.4 (*P*=0.047), VAS neck=57.9 (*P*=0.003). At 12 months, the “indifferent or worse” group showed less improvement in NDI (4.8 points, *P*<0.001) and VAS scores (2.3 points for arm pain, 4.1 points for neck pain, *P*<0.001) (see Table, Supplementary Digital Content 2, http://links.lww.com/BRS/C656, for the full detailed linear mixed model results).

**Figure 3:**
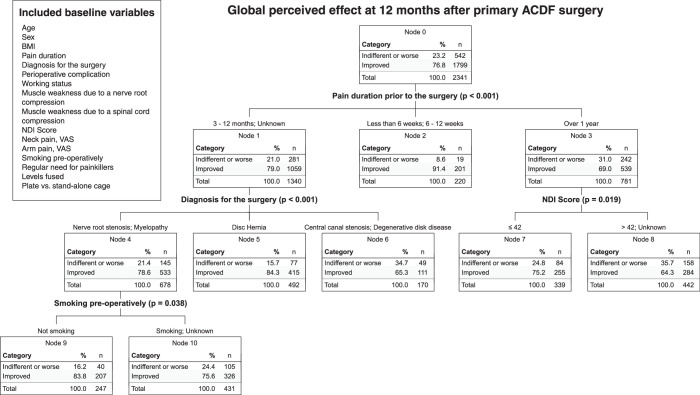
Classification tree analysis of the study population. The classification tree was built using the Chi-squared automatic interaction detection (CHAID) method. NDI Score indicates Neck Disability Index Score; VAS, visual analog scale.

Potential predictive baseline variables were entered into a classification tree analysis, resulting in three decision nodes and seven terminal nodes, illustrated in Figure 3. The most significant predictor was pain duration before surgery (*P*<0.001). Patients treated within 12 weeks of pain onset reported symptom relief in 91.4% of cases. The group with pain duration of 3 to 12 months (or unknown due to missing baseline data) had a relief rate of 78.6%. For patients whose pain duration exceeded one year, 69.0% reported relief; within this group, baseline NDI (*P*=0.019) further subdivided the group, with favorable outcomes more frequent in patients with NDI ≤42 (75.2% *vs.* 64.3%).

Within the group that had pain lasting 3 to 12 months (or unknown duration), significant differences were found based on diagnosis (*P*<0.001). Patients with disk herniation had an 84.3% improvement rate, whereas those with central canal stenosis or degenerative disk disease showed a 65.3% improvement rate. Patients with nerve root stenosis or myelopathy had an improvement rate of 78.6%, which was further subdivided by preoperative smoking status (*P*=0.038); nonsmokers were more likely to report symptom relief (83.8% *vs.* 75.6%).

Significant factors from the classification tree analysis were further analyzed using a binary logistic regression model (Table 2). Pain duration of less than one year before surgery remained the most significant predictor of symptom improvement, with an odds ratio (OR) of 1.95 (95% CI: 1.52–2.51, *P*<0.001). Preoperative NDI scores ≤42 were also associated with better outcomes (OR=1.37, 95% CI: 1.06–1.75, *P*=0.012). Nonsmokers were more likely to report symptom improvement than smokers (OR=1.37, 95% CI: 1.04–1.83, *P*=0.030). Among diagnostic groups, patients with a herniated disk were most likely to report improvement at 12 months after surgery. Patients with nerve root stenosis were the second most likely group to benefit (OR=0.61, 95% CI: 0.44–0.82, *P*<0.001), whereas patients with central canal stenosis (OR=0.42, 95% CI: 0.27–0.66, *P*<0.001) and myelopathy (OR=0.41, 95% CI: 0.21–0.85, *P*=0.007) were least likely to report symptom relief (ORs are given with disk herniation as the reference). Degenerative disk disease did not significantly affect outcomes (*P*=0.115).

**TABLE 2:** Binary Logistic Regression Model

			95% CI		
Variable	n	OR	Lower	Upper	*P*
NDI Score ≤42	690 (*vs.* 807)	**1.37**	1.06	1.75	**0.012**
Pain duration before surgery <1 y	786 (*vs.* 711)	**1.95**	1.52	2.51	**<0.001**
No smoking preoperatively	1162 (*vs.* 335)	**1.37**	1.04	1.83	**0.030**
Diagnosis					
Disk hernia (reference)	467				**<0.001**
Nerve root stenosis	784	**0.61**	0.44	0.82	**<0.001**
Central canal stenosis	178	**0.42**	0.27	0.66	**<0.001**
Myelopathy	46	**0.41**	0.21	0.85	**0.007**
Degenerative disk disease	22	0.46	0.17	1.84	0.115

Bold values are in Statistically significant *P* value.

Binary logistic regression model, internally validated by bootstrapping with 1000 resamples. Odds ratios (OR) indicate the likelihood of inclusion in the “improved” category 12 months after ACDF surgery. The included variables were selected based on the results of classification tree analysis.

NDI Score indicates Neck Disability Index Score.

## DISCUSSION

We studied predictive factors of outcome at 12 months after primary ACDF surgery in a nationwide FinSpine cohort of 5517 consecutive patients. Overall, patients benefit from ACDF surgery for degenerative cervical spine disease. Most patients report symptom relief at 12 months after surgery, with an average reduction of 23 points in NDI and 31 points in VAS arm pain compared with baseline.

Pain duration before surgery was the most substantial predictor of outcome. In our binary logistic regression model, patients whose pain lasted less than one year were almost twice as likely to report improvement at 12 months postoperatively. In the classification tree analysis, the highest GPE success rate at 12 months was observed in patients who underwent surgery within 12 weeks of symptom onset. Naturally, this group included those with the most severe symptoms—potentially introducing selection bias—yet it shows that those with severe symptoms can derive the greatest benefit from timely intervention.

Patients with lower preoperative NDI scores were also more likely to experience improvement, suggesting that less severe neck pain–related disability is associated with better overall outcomes. Smoking status had a similar impact, with nonsmokers reporting superior outcomes.

These results align with a recent publication based on the data from the Norwegian Registry for Spine Surgery (NORspine), focusing on a model of nonsuccess after cervical spine surgery for degenerative radiculopathy.^16^ In the NORspine study, pain duration over one year and NDI scores ≥41 were associated with an increased rate of nonsuccess in neck disability and arm pain at 12 months, smoking was associated with nonsuccess in arm pain only.^16^ Long-lasting pain,^14,25^ neck pain as the dominant symptom,^25^ and smoking^14,26,27^ have been found to predict poorer outcomes also in other previous studies. However, a study by Mangan *et al* ^28^ found that current smoking status did not influence PROMs at a mean follow-up time of 19.8 months.

On average, patients in our study cohort reported an NDI score in the upper range of moderate disability (30%–48%), indicating a substantial burden of neck-related symptoms. If surgery is delayed and NDI scores increase, it is reasonable to assume that comparable GPE success rates may be harder to achieve. Although high preoperative NDI scores and therefore neck pain–dominant symptoms might predict worse GPE outcomes at 12 months, ACDF surgery can still provide significant relief in neck pain, as demonstrated in Figure 2. Recent studies have also been reporting significant improvement in neck pain after ACDF.^29–31^

We found that GPE success at 12 months was also influenced by the surgical diagnosis. A significant effect was observed in the classification tree subgroup of patients with pain lasting 3 to 12 months, including those with missing baseline pain duration data. This was further confirmed by regression analysis. Patients with disk herniations showed the highest rates of improvement, followed by those with nerve root stenosis. Patients with central canal stenosis or myelopathy were least likely to report improvement, which is not unexpected, as surgery for these conditions typically aims to halt the progression of myelopathy-related symptoms.

In our study population, preoperative working status, muscle weakness caused by either nerve root compression or myelopathy, regular use of pain medication, age, gender, BMI, perioperative complications, single-level versus multilevel fusion, as well as plate versus stand-alone cage comparison, had no significant effect on GPE at 12 months in classification tree analysis. For BMI and gender, mixed results have been previously published.^14,16,32,33^ In a study of patients undergoing ACDF surgery for myelopathy or radiculopathy, Archer *et al* ^14^ found that female sex with odds of having better mJOA (modified Japanese Orthopaedic Association scale) at 12 months but worse NDI scores at 12 months. Similarly, Scerrati *et al* ^33^ found the female sex to be associated with worse NDI outcomes at 12 months. In the Scerrati *et al* ^33^ study, higher BMI correlated with worse NDI outcomes at 12 months in univariate analysis but was insignificant in multivariate analysis. Teo *et al* ^32^ reported patients exhibiting similar outcomes irrespective of BMI at six months and two years after ACDF. In the NORSpine study, gender or BMI did not predict outcomes, aligning with our results.^16^ Interestingly, perioperative complications did not significantly influence the 12-month GPE outcomes in our study. Although the evidence on ACDF outcomes is limited, existing literature on lumbar spine surgery indicates that minor complications do not significantly affect long-term outcomes.^34–36^ Given the rarity of severe complications, they are probably less likely to impact outcomes on a national scale.

A key strength of this study is the comprehensive, nationwide FinSpine register, which is specifically designed for spinal surgery and well suited to our research objectives. Finland’s nearly free public health care system ensures that all individuals have access to treatment, resulting in an unselected, representative patient sample without selection bias. Moreover, FinSpine’s coverage of all centers performing ACDF surgery generates a cohort of consecutive patients, further minimizing selection bias. This design allows the findings of the study to be generalized to a national, population-based level, presenting real-life data on ACDF surgery and factors affecting outcomes. Surgery-specific data are entered by spine surgeons themselves, meaning experts populate the register, which likely reduces the risk of errors. Furthermore, the large number of patients who completed PROMs enables robust analysis.

A common limitation in clinical register﻿ studies is incomplete data, and not all confounding factors are known. In our study, the response rate for the 12-month GPE outcome questionnaire was 42.4%, raising the possibility that loss to follow-up could introduce attrition bias. However, previous publications from Nordic national quality registers suggest that nonrespondents do not bias the outcome assessments.^37–39^ We have also recently published a study based on the current FinSpine data, demonstrating similar results.^40^ Although the outcomes do not seem to be affected by the attrition bias, patients may differ in their baseline characteristics as well as comorbidities and socioeconomic status between respondents and nonrespondents. Currently, detailed comorbidity data are not recorded in FinSpine. This omission represents a potential limitation of our study as comorbidities can significantly influence postoperative outcomes.^16^

The general limitations of retrospective studies also apply to the FinSpine register. However, our study does not involve any unique issues in this regard. In addition to attrition bias and incomplete data, we recognize the possibility of data entry errors by both patients and operating surgeons. In a national register such as FinSpine, which is populated by a large number of individuals, even specific questions can be perceived and answered differently. Therefore, harmonizing data requires validation, training, and informed communication.^22,23^

## CONCLUSIONS

This study analyzed a nationwide FinSpine cohort of 5517 patients who underwent ACDF surgery. Our findings indicate that most patients experience significant symptom relief at 12 months postsurgery. Based on these results, timely surgical intervention—specifically, operating with pain duration of less than a year and with lower Neck Disability Index (NDI) scores—appears to improve success rates as measured by PREMs/PROMs. These findings can help guide preoperative patient counseling and enhance evidence-based decision-making for treating DCSD.

Key Points

- FinSpine is a Finnish national spine register containing data on 5517 primary ACDF surgeries for degenerative cervical spine disease.
- At 12 months after surgery, 76.8% of patients reported symptom improvement.
- Shorter pain duration (≤1 yr), lower neck disability (NDI) scores (≤42), and nonsmoking were associated with better outcomes.
- Patients treated for herniated disks and nerve root stenosis were more likely to report symptom relief at 12 months compared with those with central canal stenosis or myelopathy.

## Supplementary Material

SUPPLEMENTARY MATERIAL

## References

[1] Waheed MAA Hasan S Tan LA . Cervical spine pathology and treatment: a global overview. J Spine Surg. 2020;6:340–350. 32309671 10.21037/jss.2020.01.12 PMC7154356

[2] Davies BM Phillips R Clarke D . Establishing the socio-economic impact of degenerative cervical myelopathy is fundamental to improving outcomes [AO Spine RECODE-DCM Research Priority Number 8]. Global Spine J. 2022;12:122S–129S. 35174730 10.1177/21925682211039835 PMC8859704

[3] Social insurance institution of Finland . 2023 Health insurance statistics of Finland. 2024. Accessed October 8, 2024. https://helda.helsinki.fi/items/5fd0b6c3-e4f6-4f83-a958-d46eca30597b

[4] World Health Organization . Global health estimates: Leading causes of DALYs (2000-2021). Accessed October 8, 2024. https://www.who.int/data/gho/data/themes/mortality-and-global-health-estimates/global-health-estimates-leading-causes-of-dalys

[5] Bono CM Ghiselli G Gilbert TJ . An evidence-based clinical guideline for the diagnosis and treatment of cervical radiculopathy from degenerative disorders. Spine Journal. 2011;11:64–72. 10.1016/j.spinee.2010.10.023 21168100

[6] Luyao H Xiaoxiao Y Tianxiao F . Management of cervical spondylotic radiculopathy: a systematic review. Glob Spine J. 2022;12:1912–1924. 10.1177/21925682221075290 PMC9609507 35324370

[7] Matz PG Anderson PA Holly LT . The natural history of cervical spondylotic myelopathy. J Neurosurg Spine. 2009;11:104–111. 19769489 10.3171/2009.1.SPINE08716

[8] Fehlings MG Wilson JR Kopjar B . Efficacy and safety of surgical decompression in patients with cervical spondylotic myelopathy results of the aospine north america prospective multi-center study. J Bone Jt Surg. 2013;95:1651–1658. 10.2106/JBJS.L.00589 24048552

[9] Cloward RB . The anterior approach for removal of ruptured cervical disks. J Neurosurg. 1958;15:602–617. 13599052 10.3171/jns.1958.15.6.0602

[10] Smith GW Robinson RA . The treatment of certain cervical-spine disorders by anterior removal of the intervertebral disc and interbody fusion. J Bone Joint Surg Am. 1958;40-A:607–624. 13539086

[11] Fehlings MG Barry S Kopjar B . Anterior versus posterior surgical approaches to treat cervical spondylotic myelopathy: outcomes of the prospective multicenter AOSpine north America CSM study in 264 patients. Spine (Phila Pa 1976). 2013;38:2247–2252. 24108289 10.1097/BRS.0000000000000047

[12] Wirth FP Dowd GC Sanders HF . Cervical discectomy a prospective analysis of three operative techniques. Surgical Neurology. 2000;53:340–346. 10825519 10.1016/s0090-3019(00)00201-9

[13] Peolsson A Hedlund R Vavruch L . Predictive factors for the outcome of anterior cervical decompression and fusion. Eur Spine J. 2003;12:274–280. 12687444 10.1007/s00586-003-0530-2 PMC3615504

[14] Archer KR Bydon M Khan I . Development and validation of cervical prediction models for patient-reported outcomes at 1 year after cervical spine surgery for radiculopathy and myelopathy. Spine (Phila Pa 1976). 2020;45:1541–1552. 32796461 10.1097/BRS.0000000000003610

[15] Hermansen A Hedlund R Vavruch L . Positive predictive factors and subgroup analysis of clinically relevant improvement after anterior cervical decompression and fusion for cervical disc disease: A 10- to 13-year follow-up of a prospective randomized study. J Neurosurg Spine. 2013;19:403–411. 23909550 10.3171/2013.7.SPINE12843

[16] Mjåset C Solberg TK Zwart JA . Anterior surgical treatment for cervical degenerative radiculopathy: a prediction model for non-success. Acta Neurochir (Wien). 2023;165:145–157. 36481873 10.1007/s00701-022-05440-2 PMC9840586

[17] Marjamaa J Huttunen J Kankare J . The Finnish spine register (FinSpine): development, design, validation and utility. Eur Spine J. 2023;32:3731–3743. 37718342 10.1007/s00586-023-07874-3

[18] Widbom-Kolhanen S Pernaa KI Saltychev M . Reliability and validity of the Neck Disability Index among patients undergoing cervical surgery. Int J Rehabil Res. 2022;45:273–278. 35776945 10.1097/MRR.0000000000000540

[19] Fairbank JCT Pynsent PB . The Oswestry Disability Index. Spine (Phila Pa 1976). 2000;25:2940–2953. 11074683 10.1097/00007632-200011150-00017

[20] EuroQol Group . EuroQol - a new facility for the measurement of health-related quality of life. Health Policy (New York). 1990;16:199–208. 10.1016/0168-8510(90)90421-9 10109801

[21] Kamper SJ Ostelo RWJG Knol DL . Global Perceived Effect scales provided reliable assessments of health transition in people with musculoskeletal disorders, but ratings are strongly influenced by current status. J Clin Epidemiol. 2010;63:760–766.e1. 20056385 10.1016/j.jclinepi.2009.09.009

[22] Hooff MLV Jacobs WCH Willems PC . Evidence and practice in spine registries. Acta Orthop. 2015;86:534–544. 25909475 10.3109/17453674.2015.1043174 PMC4564774

[23] Pascucci S Langella F Franzò M . National spine surgery registries’ characteristics and aims: globally accepted standards have yet to be met. Results of a scoping review and a complementary survey. J Orthop Traumatol. 2023;24:49. 37715871 10.1186/s10195-023-00732-4 PMC10505129

[24] Röder C Müller U Aebi M . The rationale for a spine registry. Eur Spine J. 2006;15:S52–S56. 16292634 10.1007/s00586-005-1050-z PMC3454550

[25] Divi SN Goyal DKC Woods BI . How do patients with predominant neck pain improve after anterior cervical discectomy and fusion for cervical radiculopathy? Int J Spine Surg. 2022;16:240–246. 35273114 10.14444/8212 PMC9930673

[26] Davis RJ Kim KD Hisey MS . Cervical total disc replacement with the mobi-C cervical artificial disc compared with anterior discectomy and fusion for treatment of 2-level symptomatic degenerative disc disease: a prospective, randomized, controlled multicenter clinical trial. Clinical article J Neurosurg Spine. 2013;19:532–545. 10.3171/2013.6.SPINE12527 24010901

[27] Hilibrand AS Fye MA Emery SE . Impact of smoking on the outcome of anterior cervical arthrodesis with interbody or strut-grafting. J Bone Joint Surg Am. 2001;83:668–673. 11379735 10.2106/00004623-200105000-00004

[28] Mangan JJ Goyal DKC Divi SN . Does smoking status influence health-related ouality of life outcome measures in patients undergoing ACDF? Global Spine J. 2021;11:50–56. 32875848 10.1177/2192568219890292 PMC7734264

[29] Li Y Yang L Wu Y . Anterior cervical decompression and fusion for neck pain in patients with cervical spondylosis: pooled results of three prospective cohort studies. World Neurosurg. 2024;190:e694–e700. 39111662 10.1016/j.wneu.2024.07.208

[30] Massel DH Mayo BC Bohl DD . Improvements in neck and arm pain following an anterior cervical discectomy and fusion. Spine (Phila Pa 1976). 2017;42:E825–E832. 27851659 10.1097/BRS.0000000000001979

[31] Stull JD Goyal DKC Mangan JJ . The outcomes of patients with neck pain following ACDF: a comparison of patients with radiculopathy, myelopathy, or mixed symptomatology. Spine (Phila Pa 1976). 2020;45:1485–1490. 32796460 10.1097/BRS.0000000000003613

[32] Teo SJ Ling MZ Fong PL . The effect of body mass index on long-term patient-reported outcome scores after anterior cervical discectomy and fusion in an Asian population: a 2-year study. Asian Spine J. 2021;15:512–522. 32951406 10.31616/asj.2020.0012 PMC8377209

[33] Scerrati A Germano’ A Montano N . Factors affecting functional outcome after anterior cervical discectomy and fusion: a multicenter study. J Craniovertebr Junction Spine. 2021;12:144–148. 34194160 10.4103/jcvjs.jcvjs_1_21 PMC8214232

[34] Strömqvist F Sigmundsson FG Strömqvist B . Incidental durotomy in degenerative lumbar spine surgery—a register study of 64,431 operations. Spine J. 2019;19:624–630. 30172899 10.1016/j.spinee.2018.08.012

[35] Ayling OGS Ailon T Street JT . The effect of perioperative adverse events on long-term patient-reported outcomes after lumbar spine surgery. Neurosurgery. 2021;88:420–427. 33009559 10.1093/neuros/nyaa427

[36] Lambat MP Glassman SD Carreon LY . Impact of perioperative complications on clinical outcome scores in lumbar fusion surgery. J Neurosurg Spine. 2013;18:265–268. 23289509 10.3171/2012.12.SPINE12805

[37] Højmark K Støttrup C Carreon L . Patient-reported outcome measures unbiased by loss of follow-up. Single-center study based on DaneSpine, the Danish spine surgery registry. Eur Spine J. 2016;25:282–286. 26208938 10.1007/s00586-015-4127-3

[38] Ingebrigtsen T Aune G Karlsen ME . Non-respondents do not bias outcome assessment after cervical spine surgery: a multicenter observational study from the Norwegian registry for spine surgery (NORspine). Acta Neurochir (Wien). 2023;165:125–133. 36539647 10.1007/s00701-022-05453-x PMC9840578

[39] Elkan P Lagerbäck T Möller H . Response rate does not affect patient-reported outcome after lumbar discectomy. Eur Spine J. 2018;27:1538–1546. 29523985 10.1007/s00586-018-5541-0

[40] Klimko N Danner N Salo H . Outcome measures after anterior cervical decompression and fusion surgery—non-respondents do not bias the results: a Finnish spine register (FinSpine) study. Brain and Spine. 2025;5:104179 10.1097/BRS.0000000000005323 PMC12011435 40047144

