# Supplementary material for: Outcome After Anterior Cervical Decompression and Fusion—A Nationwide FinSpine Register Study of Independent Predictors of Outcome at 12 Months After Surgery for Degenerative Cervical Spine
Source: Spine (Phila Pa 1976). 2025 Mar 6;50(10):664–71. doi: 10.1097/BRS.0000000000005323 (PMC12011435; doi:10.1097/BRS.0000000000005323)
Supplement: SUPPLEMENTARY MATERIAL [file brs-50-664-s001.docx]

| **Symptom status at 12 mo post-op** | **Time** | **count** | **mean** | **median** | **StdErr** | **IQR** | **min** | **max** |
| --- | --- | --- | --- | --- | --- | --- | --- | --- |
| Indifferent or worse | Baseline | 368 | 45.47 | 46 | 17.18 | 24.00 | 0 | 88 |
| Indifferent or worse | 3 Months | 366 | 35.13 | 34 | 18.74 | 27.50 | 0 | 80 |
| Indifferent or worse | 12 Months | 503 | 41.64 | 42 | 18.55 | 26.00 | 0 | 90 |
| Improved | Baseline | 1,206 | 41.69 | 42 | 16.80 | 23.00 | 0 | 96 |
| Improved | 3 Months | 1,104 | 20.35 | 16 | 16.20 | 22.00 | 0 | 86 |
| Improved | 12 Months | 1,640 | 18.79 | 16 | 15.80 | 22.00 | 0 | 82 |
| Non-GPE PROM data only | Baseline | 1,343 | 42.82 | 44 | 17.25 | 23.00 | 0 | 92 |
| Non-GPE PROM data only | 3 Months | 920 | 24.24 | 22 | 17.88 | 26.00 | 0 | 84 |
| Non-GPE PROM data only | 12 Months | 112 | 28.38 | 24 | 18.66 | 29.75 | 0 | 65 |

**NDI descriptives**

**VAS Neck descriptives**

| **Symptom status at 12 mo post-op** | **Time** | **count** | **mean** | **median** | **StdErr** | **IQR** | **min** | **max** |
| --- | --- | --- | --- | --- | --- | --- | --- | --- |
| Indifferent or worse | Baseline | 373 | 58.13 | 65.0 | 26.75 | 34.0 | 0 | 100 |
| Indifferent or worse | 3 Months | 360 | 40.89 | 39.5 | 27.70 | 48.0 | 0 | 100 |
| Indifferent or worse | 12 Months | 527 | 53.77 | 62.0 | 27.24 | 44.0 | 0 | 100 |
| Improved | Baseline | 1,222 | 53.42 | 60.0 | 27.41 | 43.0 | 0 | 100 |
| Improved | 3 Months | 1,053 | 24.38 | 16.0 | 24.20 | 32.0 | 0 | 100 |
| Improved | 12 Months | 1,624 | 24.34 | 16.0 | 24.43 | 36.0 | 0 | 100 |
| Non-GPE PROM data only | Baseline | 1,328 | 56.67 | 64.0 | 26.39 | 40.0 | 0 | 100 |
| Non-GPE PROM data only | 3 Months | 903 | 28.03 | 20.0 | 25.88 | 43.5 | 0 | 94 |
| Non-GPE PROM data only | 12 Months | 130 | 32.91 | 27.0 | 28.43 | 54.5 | 0 | 93 |

| **Symptom status at 12 mo post-op** | **Time** | **count** | **mean** | **median** | **StdErr** | **IQR** | **min** | **max** |
| --- | --- | --- | --- | --- | --- | --- | --- | --- |
| Indifferent or worse | Baseline | 366 | 58.63 | 64.0 | 26.77 | 36.75 | 0 | 100 |
| Indifferent or worse | 3 Months | 350 | 41.44 | 43.5 | 29.98 | 55.00 | 0 | 100 |
| Indifferent or worse | 12 Months | 523 | 56.10 | 64.0 | 27.62 | 42.00 | 0 | 100 |
| Improved | Baseline | 1,193 | 55.33 | 61.0 | 27.96 | 46.00 | 0 | 100 |
| Improved | 3 Months | 1,004 | 23.88 | 14.0 | 25.98 | 39.00 | 0 | 100 |
| Improved | 12 Months | 1,561 | 24.67 | 14.0 | 26.21 | 41.00 | 0 | 100 |
| Non-GPE PROM data only | Baseline | 1,301 | 59.23 | 66.0 | 26.37 | 38.00 | 0 | 100 |
| Non-GPE PROM data only | 3 Months | 878 | 28.41 | 18.0 | 28.22 | 50.00 | 0 | 100 |
| Non-GPE PROM data only | 12 Months | 127 | 37.38 | 34.0 | 29.27 | 55.00 | 0 | 100 |

**VAS Arm descriptives**

“Non-GPE PROM data only” -group represents a group of patients for whom the Global perceived effect (GPE) outcome variable data was lost to follow-up. VAS = Visual analogue scale, NDI Score = Neck disability index score, StdErr = Standard error of the mean, IQR = Interquartile range
