# Supplementary material for: Outcome After Anterior Cervical Decompression and Fusion—A Nationwide FinSpine Register Study of Independent Predictors of Outcome at 12 Months After Surgery for Degenerative Cervical Spine
Source: Spine (Phila Pa 1976). 2025 Mar 6;50(10):664–71. doi: 10.1097/BRS.0000000000005323 (PMC12011435; doi:10.1097/BRS.0000000000005323)
Supplement: SUPPLEMENTARY MATERIAL [file brs-50-664-s002.docx]

|  |  |  |  |  | **95% C.I.** | |
| --- | --- | --- | --- | --- | --- | --- |
| **Description** | **Estimate** | **StdErr** | **t Value** | **P-value** | **Lower** | **Upper** |
| Baseline NDI score for "Improved". | **41.78** | 0.45 | 92.02 | **< 0.001** | 40.89 | 42.67 |
| Change in NDI score at 3 months for "Improved". | **-21.27** | 0.47 | -45.23 | **< 0.001** | -22.19 | -20.35 |
| Change in NDI score at 12 months for "Improved". | **-23.02** | 0.42 | -54.49 | **< 0.001** | -23.85 | -22.20 |
| Difference in baseline NDI score for "Indifferent or worse" vs. "Improved". | **4.58** | 0.94 | 4.89 | **< 0.001** | 2.75 | 6.42 |
| Additional change in NDI score at 3 months for Improved" vs. "Indifferent or worse”. | **-10.28** | 0.95 | -10.80 | **< 0.001** | -12.15 | -8.41 |
| Additional change in NDI score at 12 months for Improved" vs. "Indifferent or worse”. | **-18.20** | 0.87 | -20.87 | **< 0.001** | -19.91 | -16.49 |

**NDI Score Linear mixed model results**

|  |  |  |  |  | **95% C.I.** | |
| --- | --- | --- | --- | --- | --- | --- |
| **Description** | **Estimate** | **StdErr** | **t Value** | **P-value** | **Lower** | **Upper** |
| Baseline VAS score for "Improved". | **53.41** | 0.72 | 73.99 | **< 0.001** | 52.00 | 54.83 |
| Change in VAS score at 3 months for "Improved". | **-29.37** | 0.92 | -32.06 | **< 0.001** | -31.16 | -27.57 |
| Change in VAS score at 12 months for "Improved". | **-29.19** | 0.82 | -35.71 | **< 0.001** | -30.79 | -27.59 |
| Difference in baseline VAS score for "Indifferent or worse" vs. "Improved". | **4.52** | 1.49 | 3.03 | **0.0025** | 1.60 | 7.44 |
| Additional VAS reduction at 3 months for "Improved" vs. "Indifferent or worse" | **-11.63** | 1.84 | -6.31 | **< 0.001** | -15.24 | -8.01 |
| Additional VAS reduction at 12 months for "Improved" vs. "Indifferent or worse" | **-25.10** | 1.66 | -15.08 | **< 0.001** | -28.36 | -21.84 |

**VAS Neck Linear mixed model results**

**VAS Arm Linear mixed model results**

|  |  |  |  |  | **95% C.I.** | |
| --- | --- | --- | --- | --- | --- | --- |
| **Description** | **Estimate** | **StdErr** | **t Value** | **P-value** | **Lower** | **Upper** |
| Baseline VAS score for "Improved". | **55.22** | 0.77 | 72.07 | **< 0.001** | 53.72 | 56.72 |
| Change in VAS score at 3 months for "Improved". | **-31.48** | 0.98 | -32.04 | **< 0.001** | -33.41 | -29.56 |
| Change in VAS score at 12 months for "Improved". | **-30.65** | 0.87 | -35.11 | **< 0.001** | -32.37 | -28.94 |
| Difference in baseline VAS score for "Indifferent or worse" vs. "Improved". | **3.14** | 1.58 | 1.99 | **0.0470** | 0.04 | 6.23 |
| Additional VAS reduction at 3 months for "Improved" vs. "Indifferent or worse" | **-14.32** | 1.96 | -7.29 | **< 0.001** | -18.17 | -10.46 |
| Additional VAS reduction at 12 months for "Improved" vs. "Indifferent or worse" | **-28.37** | 1.77 | -16.05 | **< 0.001** | -31.84 | -24.91 |

Linear mixed model results representing differences in the Global Perceived Effect variable between groups, as well as within groups, over time. VAS = Visual analogue scale, NDI Score = Neck disability index score, StdErr = Standard error of the mean, 95% C.I. = 95% confidence interval.
